# Synthesis and enhanced photocatalytic performance of 0D/2D CuO/tourmaline composite photocatalysts

**DOI:** 10.3762/bjnano.11.31

**Published:** 2020-03-02

**Authors:** Changqiang Yu, Min Wen, Zhen Tong, Shuhua Li, Yanhong Yin, Xianbin Liu, Yesheng Li, Tongxiang Liang, Ziping Wu, Dionysios D Dionysiou

**Affiliations:** 1 School of Materials Science and Engineering, Jiangxi University of Science and Technology, Ganzhou 341000, China; 2 Environmental Engineering and Science Program, Department of Chemical and Environmental Engineering, University of Cincinnati, Cincinnati, OH 45221, USA; 3 Chongyi Zhangyuan Tungsten Co., Ltd., Ganzhou 341000, China

**Keywords:** 0D/2D CuO, organic contaminants, photocatalytic activity, photoinduced charge separation, tourmaline

## Abstract

Photocatalysis is considered to be a green and promising technology for transforming organic contaminants into nontoxic products. In this work, a CuO/tourmaline composite with zero-dimensional/two-dimensional (0D/2D) CuO architecture was successfully obtained via a facile hydrothermal process, and its photocatalytic activity was evaluated by the degradation of methylene blue (MB). Surface element valence state and molecular vibration characterization revealed that CuO chemically interacted with tourmaline via Si–O–Cu bonds. The specific surface area of the CuO/tourmaline composite (23.60 m^2^ g^−1^) was larger than that of the pristine CuO sample (3.41 m^2^ g^−1^). The CuO/tourmaline composite exhibited excellent photocatalytic activity for the degradation of MB, which was ascribed to the increase in the quantity of the adsorption-photoreactive sites and the efficient utilization of the photoinduced charge carriers. This study provides a facile strategy for the construction of 0D/2D CuO structures and the design of tourmaline-based functional composite photocatalysts for the treatment of organic contaminants in water.

## Introduction

Developing a novel semiconductor with excellent photoreactive activity toward the treatment of organic contaminants in wastewater is in urgent need owing to the deterioration of the ecological environment [1]. Metal oxides, such as ZnO [2], TiO_2_ [3], Fe_2_O_3_ [4], and CuO [5], have been demonstrated to be promising photocatalysts. In particular, the band gap energy (*E*_g_) of the p-type CuO semiconductor is ≈1.2 eV [6]. The application of CuO covers the fields of photocatalytic degradation of organic contaminants [7], photocatalytic reduction of CO_2_ [8–9], photocatalytic splitting of water [10], etc. Nanoscale CuO has been widely studied owing to its increased surface defects and higher specific surface area compared with its bulk counterpart [11–12]. However, the agglomeration of nanoscale CuO results from the high surface energy and the quick recombination of the photoinduced charge carriers and restricts the photocatalytic activity [13–14]. At present, the construction of CuO-based heterostructures (e.g., 0D/2D CuO/TiO_2_, 0D/3D CuO/ZnO, 2D/2D CuO/Fe_2_O_3_, 0D/2D CuO/C_3_N_4_, 2D/0D CuO/Ag_3_PO_4_) [6,12,15–17] and the dispersion of CuO on supporting materials (e.g., graphene, carbon nanotube) [7,18] are considered to be the most effective ways to address these problems. However, it is difficult to obtain uniform heterostructures, and the preparation process is either complex or of high cost. Therefore, exploring low-cost and easily synthesized CuO-based materials with good photoreactive properties is still an urgent task. Especially the 0D/2D structure has been proven to be effective for enhancing the photocatalytic activity due to the decrease in the distance for the separation and migration of charge carriers [19] and the introduction of nanoparticles [20].

Tourmaline is a type of natural polar mineral, and its general formula can be written as *XY*_3_*Z*_6_(*T*_6_O_18_)(*B*O_3_)_3_*V*_3_*W*, where *X*: K^+^, Na^+^, Ca^2+^; *Y*: Li^+^, Fe^2+^, Mn^2+^, Mg^2+^, Fe^3+^, Al^3+^, Cr^3+^, V^3+^, Ti^4+^; *Z*: Fe^3+^, Al^3+^, Cr^3+^, V^3+^; *T*: Al^3+^, B^3+^, Si^4+^; *B*: B^3+^; *V*: OH^−^, O^2−^; *W*: OH^−^, F^−^, O^2−^ [21]. The spontaneous permanent polarization provides tourmaline with an electric field of 10^6^–10^7^ V m^−1^ on its surface [22]. The electric field can increase the separation of the photoinduced charge carriers [2,23–24]. Furthermore, tourmaline can augment the oxygen dissolved into water due to its infrared radiation effect [24–25], which contributes to accelerate the photocatalytic oxidation reactions. Tourmaline is a promising functional mineral material for accepting the photogenerated e^−^ due to its special electric field.

Herein, we report the successful synthesis of the CuO/tourmaline composite photocatalyst with 0D/2D CuO geometric structure by a facile precipitation–hydrothermal process. This work firstly highlights a simple way to promote the photocatalytic activity of CuO by coupling with the polar mineral tourmaline, and provides an ideal example for the development of easily synthesized and low-cost tourmaline-based photocatalysts. The morphology, microstructure, pore structure, optical properties, and durability of the samples were characterized and discussed. The photocatalytic degradation performance of the CuO/tourmaline composite was investigated, and a plausible mechanism was proposed.

## Experimental

### Materials

The tourmaline sample used in this study was obtained from Hebei province, China. It was crushed and purified through a hand-selecting process. Then, distilled water was used to rinse the tourmaline sample, and the obtained sample was dried at 110 °C. Finally, the obtained sample was ground by a planetary mill and filtered (325 mesh) for use in the experiments.

Cupric acetate monohydrate (Cu(CH_3_COO)_2_·H_2_O, AR), sodium hydroxide (NaOH, AR), sodium sulfite (Na_2_SO_3_, AR), sodium sulfide (Na_2_S, AR), 5,5-dimethyl-1-pyrroline *N*-oxide (DMPO, AR), disodium ethylenediaminetetraacetate (EDTA, AR), *tert*-butyl alcohol (TBA, AR), 1,4-benzoquinone (BQ, AR), and Nafion were purchased from Aladdin Chemistry Co., Ltd., China.

### Photocatalyst synthesis

A hydrothermal process was employed to synthesize the CuO/tourmaline composite. In detail, 0.50 g of the tourmaline powder and 40 mL of distilled water were added into a beaker followed by 20 min of magnetic stirring, and the obtained suspension was sonicated for 20 min to ensure the formation of a homogeneous suspension A. Then, 1.25 g of Cu(CH_3_COO)_2_·H_2_O was dissolved into suspension A to form suspension B. A homogeneous solution C was obtained by dissolving 0.50 g of NaOH into 20 mL of distilled water, and it was mixed dropwise with suspension B with vigorous stirring for 30 min. Finally, the as-formed suspension was transferred into a polytetrafluoroethylene reactor with a total volume of 100 mL and kept at 180 °C for 6 h. After naturally cooling down, deionized water was used to wash the resultant products and the obtained samples were dried under 70 °C, and the product was named CuO/tourmaline-1:1 (1:1 in mass ratio of the CuO/tourmaline). By controlling the dosage of Cu(CH_3_COO)_2_·H_2_O and NaOH in proportion the CuO/tourmaline composites in different mass ratios of the CuO/tourmaline were acquired.

### Characterization

X-ray diffraction (XRD) patterns were obtained using step scan mode performed with a DX-2700 diffractometer (Dandong Haoyuan Instrument Co. Ltd., China) with Ni-filtered Cu Kα radiation (λ = 0.1.541 Å), 10–80° 2θ scan. X-ray photoelectron spectra (XPS) were recorded using a K-ALPHA instrument (ThermoFisher Scientific, USA). A MIRA3 field emission scanning electron microscope (SEM, Tescan, Czech) was used to observe the morphology. A Titan G2 60-300 transmission electron microscope (TEM, FEI, USA) was employed to analyze the microstructure. A NEXUS 670 spectrometer (Thermo Nicolet, USA) was employed for recording the Fourier transform infrared (FTIR) spectra. UV–visible diffuse reflectance spectra were measured using a Lambda 750S UV–vis spectrophotometer (Perkin-Elmer, USA) calibrated using barium sulfate. Photoluminescence (PL) spectra were measured with a F-4600 fluorescence spectrophotometer (Hitachi, Japan) with an excitation wavelength of 400 nm. The transient photocurrent was measured with two Interface 1010E electrochemical workstations (Gamry, USA) using Na_2_SO_3_ (0.2 M) and Na_2_S (0.2 M) solutions with a volume ratio of 1:1 as electrolyte under LED lamp (λ = 470 nm) irradiation for 20 min. The samples were prepared as follows: Firstly, 10 mg of the samples were mixed with 2 mL of ethanol followed by 10 min of sonication. Then, 50 µL of 5% Nafion were added, and the obtained mixture was magnetically stirred for 24 h. Finally, the mixture was coated on ITO glass and dried in a vacuum oven for 12 h. Electron paramagnetic resonance (EPR) spectra were analyzed using an A300 spectrometer (Bruker, Germany) with DMPO as a free radical scavenger under visible light irradiation (λ = 420 nm) for 20 min. The surface properties were characterized via an ASAP 2020 instrument (Micromeritics, USA). The zeta potential was measured with a ZETASIZER NANO ZS90 instrument (Malvern, UK).

### Photocatalytic activity evaluation

The photocatalytic performance was examined by the degradation of MB with light irradiation simulated by a 300 W Xe lamp with a 420 nm cut-off filter. In detail, 0.05 g of the as-synthesized photocatalyst was added into 100 mL of 0.01 g L^−1^ MB aqueous solution, followed by 1 h of magnetic stirring in the dark to make sure the adsorption saturation of MB onto the photocatalysts was achieved. Then, the photocatalyst–dye aqueous suspension system was exposed to light. 4 mL of the aqueous suspension was extracted every 20 min and clarified by centrifugation for the measurement of absorbance, which can be used to monitor the progress of the photocatalytic degradation reactions. For the durability test, the used photocatalyst was firstly rinsed with ethanol and deionized water by centrifugation, then it was collected by using aqueous MB for the subsequent experiments.

## Results and Discussion

**Figure 1:**
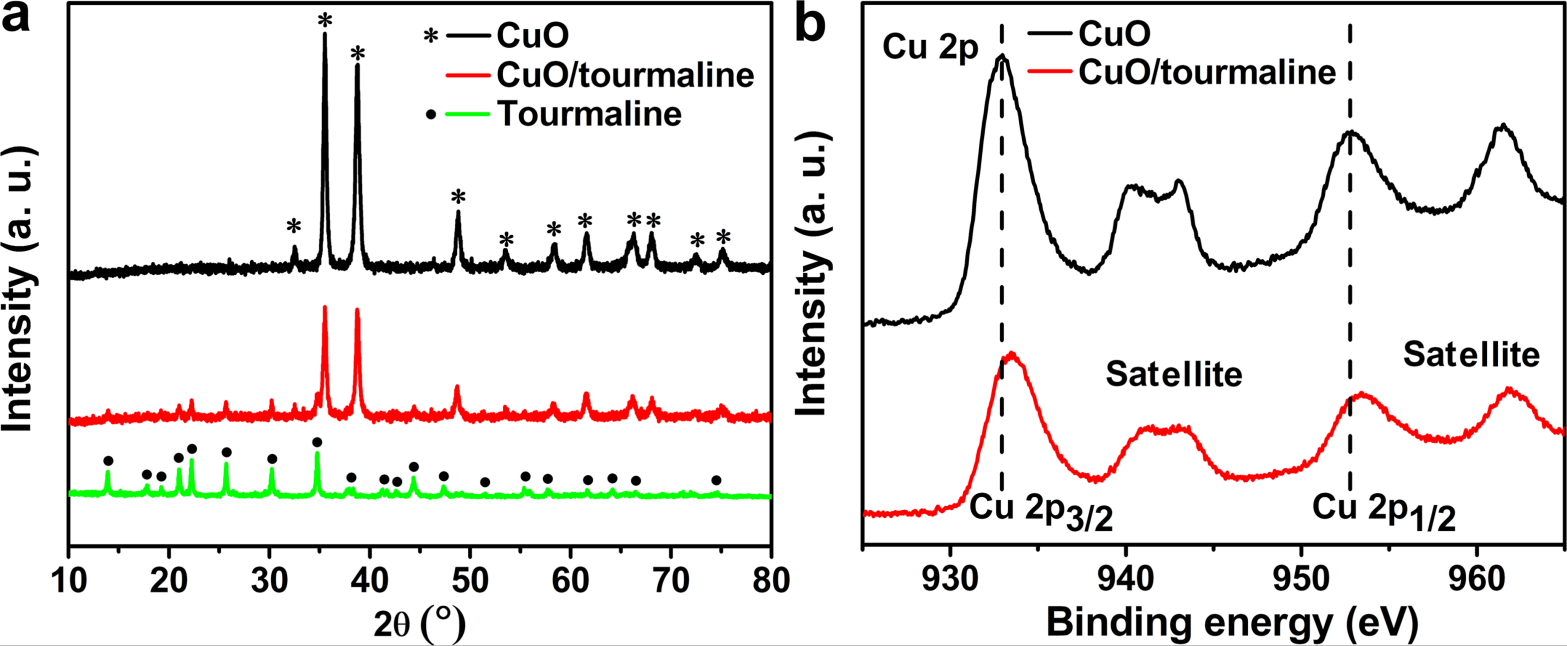
(a) XRD spectra of the CuO, tourmaline, and CuO/tourmaline composite. (b) High-resolution Cu 2p XPS spectra of the CuO and CuO/tourmaline composite.

Figure 1a displays the XRD spectra of the samples. The main peaks with 2θ values of 32.51°, 35.54°, 38.71°, 48.72°, 53.49°, 58.26°, 61.52°, 66.22°, 68.12°, 72.37°, and 75.24° matched well with those of monoclinic CuO (JCPDS 48-1548). As for tourmaline, the main diffraction peaks with 2θ values of 13.92°, 17.82°, 19.24°, 21.02°, 22.25°, 25.68°, 29.57°, 30.23°, 34.74°, 37.86°, 38.32°, 41.21°, 41.64°, 42.62°, 44.35°, 47.32°, 48.59°, 55.38°, 55.85°, 57.69°, 61.69°, 64.06°, and 74.56° were assigned to the (101), (021), (300), (211), (220), (012), (410), (122), (051), (232), (511), (502), (431), (303), (152), (342), (143), (063), (271), (550), (054), (713), and (505) planes of schorl (JCPDS 85-1811). No impurity peaks were detected, indicating the predominance of CuO and tourmaline in the composite.

The surface element component and valence state of the as-synthesized photocatalysts were investigated with XPS (Figure 1b). Two XPS peaks with binding energy of 932.93 eV and 952.79 eV correspond to Cu 2p_3/2_ and Cu 2p_1/2_, respectively. The satellite peaks with higher binding energy of 941.78 eV and 961.45 eV were observed [26]. In comparison to pure CuO, the binding energy of Cu 2p_3/2_ and Cu 2p_1/2_ for the CuO/tourmaline composite was shifted to 933.49 eV and 953.37 eV, respectively, providing evidence of the chemical interaction between CuO and tourmaline.

**Figure 2:**
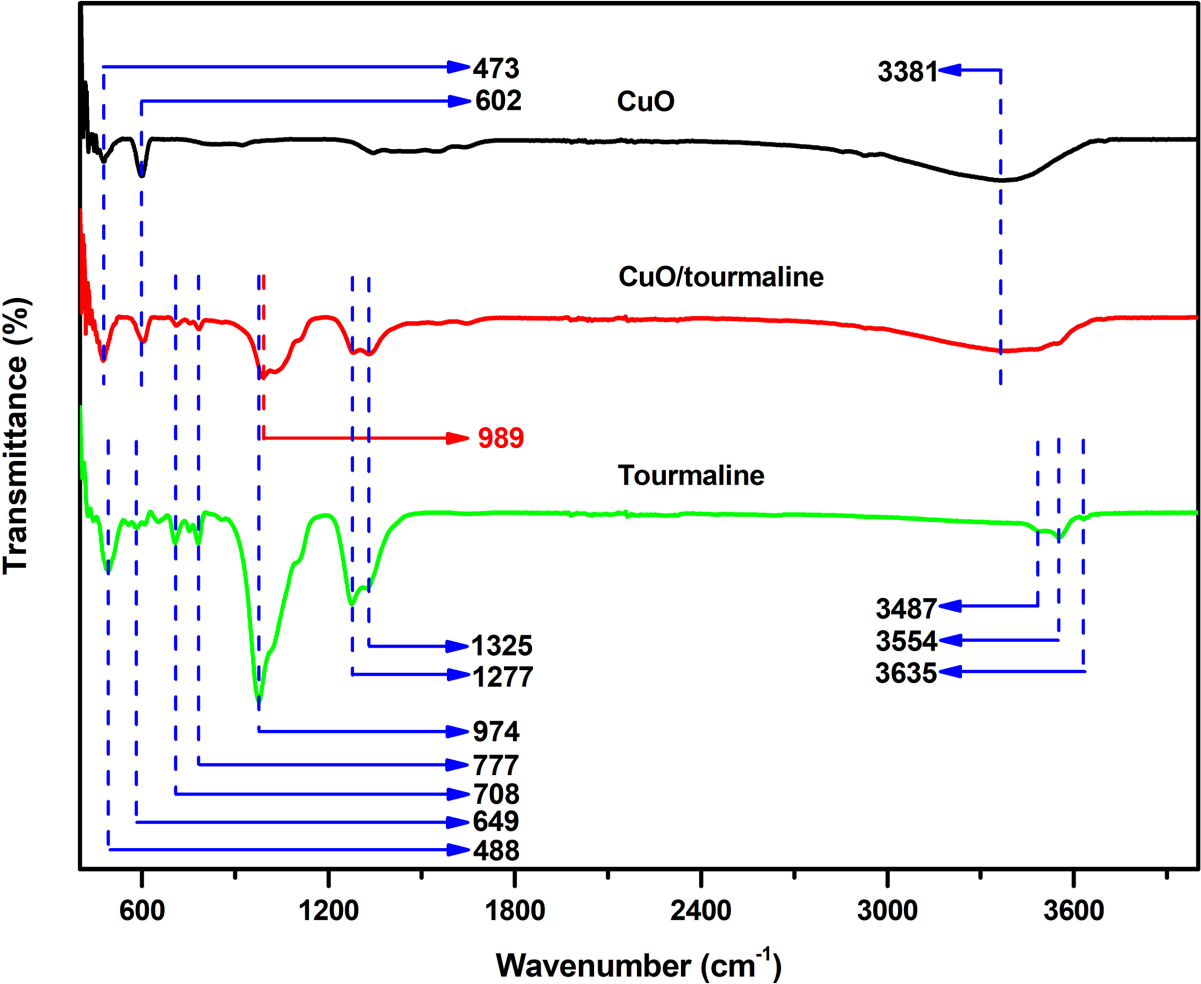
FTIR spectra of the CuO, tourmaline, and CuO/tourmaline composite.

It is convenient to detect the chemical bonds in complex materials by FTIR spectroscopy. As shown in Figure 2, three bands appeared at 3487 cm^−1^, 3554 cm^−1^, and 3635 cm^−1^ resulting from the vibration of the three OH groups in tourmaline [2]. The bending vibration of the Si–O group was detected at 488 cm^−1^ [27]. The band at 649 cm^−1^ was due to the *R*^IV^–O (*R* = Al, Fe, Mg, Mn) stretching vibration, and the peaks at 708 cm^−1^ and 777 cm^−1^ result from the Si–O–Si stretching vibration [2,28]. A well-resolved band at 974 cm^−1^ originated from the Si–O stretching vibration [29], and was shifted to 989 cm^−1^ with a blue-shift of 15 cm^−1^ for the CuO/tourmaline composite compared with that of the pristine tourmaline. This result indicated that tourmaline could chemically interact with CuO via Si–O–Cu bonds. The two bands at 1277 cm^−1^ and 1325 cm^−1^ came from the planar three-fold boron stretching vibration. As for CuO, the peaks at 473 cm^−1^ and 602 cm^−1^ resulted from the vibration of the Cu–O group [30]. In addition, the O–H stretching vibration band of the absorbed water appeared at 3381 cm^−1^ [31].

**Figure 3:**
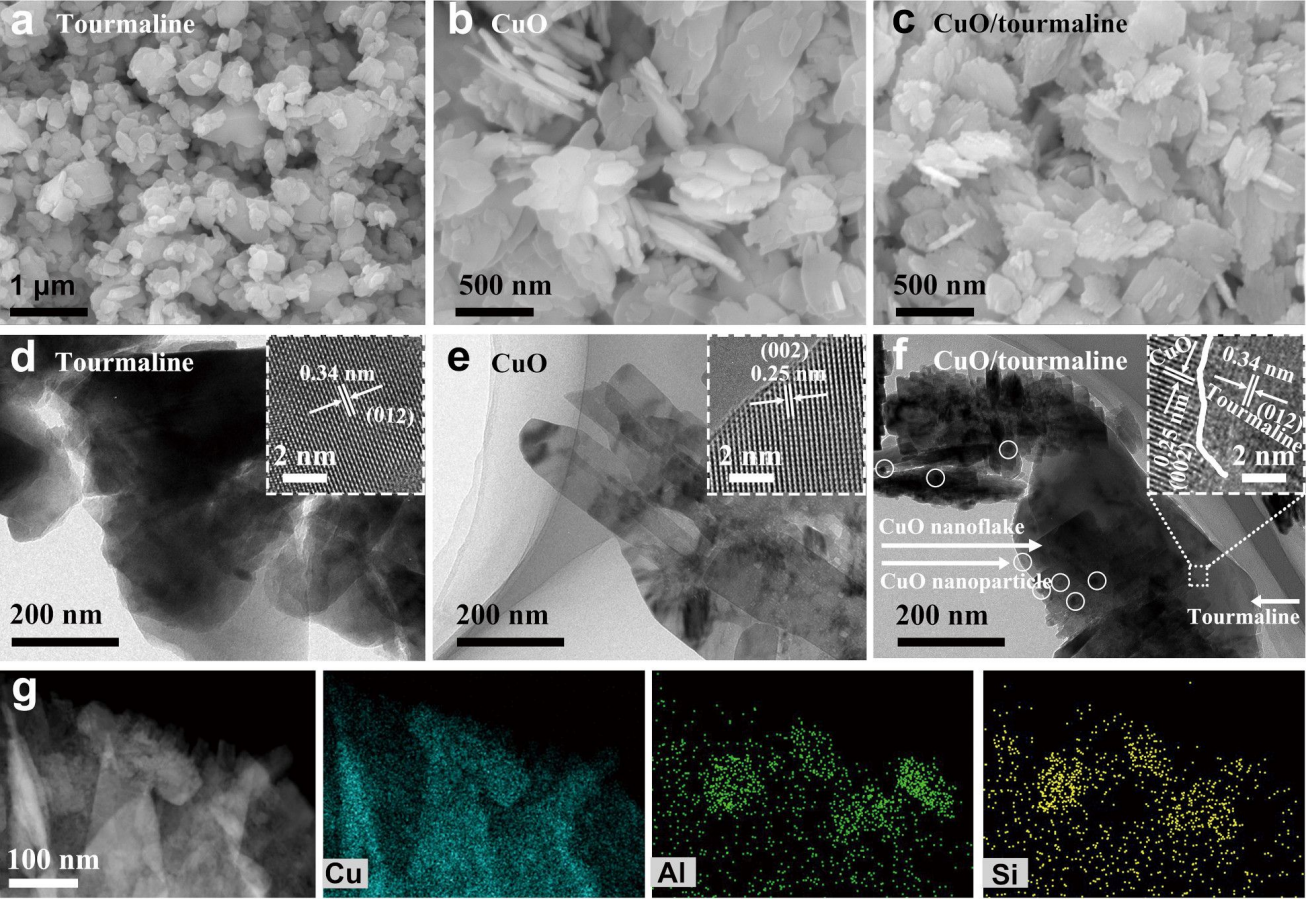
SEM images of (a) tourmaline, (b) CuO, and (c) CuO/tourmaline composite. TEM images of (d) tourmaline, (e) CuO, and (f) CuO/tourmaline composite, where the insets show the corresponding high-resolution TEM images. (g) EDX element mapping images of Cu, Al, and Si for the CuO/tourmaline composite.

SEM and TEM were carried out to characterize the morphology and microstructure of the samples. Tourmaline displayed a granular morphology with diameter of 0.1–1.0 μm (Figure 3a and 3d). The pure CuO was composed of highly stacked smooth 2D nanoflakes with a thickness of ≈50 nm (Figure 3b and 3e). With the addition of tourmaline, plenty of 0D CuO nanoparticles (≈20 nm) were generated on the surface of 2D CuO nanoflakes (Figure 3c and 3f). The 0D CuO nanoparticles roughened the surface of the 2D CuO nanoflakes and significantly alleviated their agglomeration. The lattice fringes with a *d* spacing of 0.34 nm and 0.25 nm matched well with the (012) plane of tourmaline and the (002) plane of CuO, respectively (Figure 3d,e, inset). There was intimate interfacial contact between CuO and tourmaline (Figure 3f, inset), which provided channels for the fast transfer of photoinduced e^−^ from the conduction band (CB) of CuO to tourmaline. The energy dispersive X-ray (EDX) elemental mapping verified the uniform dispersion of CuO throughout the CuO/tourmaline composite (Figure 3g).

**Figure 4:**
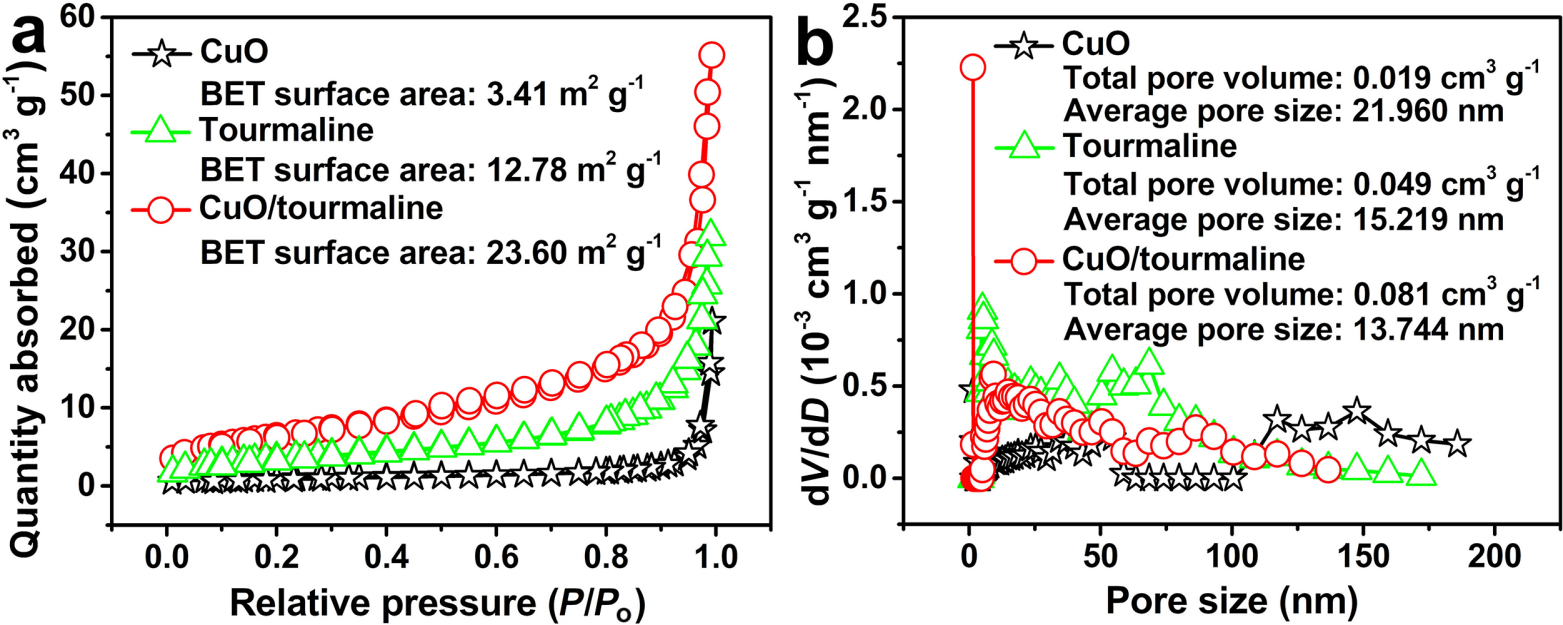
(a) Nitrogen adsorption–desorption isotherms and (b) BJH pore size distribution of the CuO, tourmaline, and CuO/tourmaline composite.

The pore structure of the photocatalysts was analyzed by their nitrogen adsorption–desorption isotherms. As shown in Figure 4a, CuO, tourmaline, and CuO/tourmaline composite exhibited type-IV adsorption isotherms, which are characteristic of mesoporous materials. As shown in Table 1, the Brunauer–Emmett–Teller (BET) specific surface area of the CuO/tourmaline composite with 0D/2D CuO structure (23.60 m^2^ g^−1^) was larger than that of CuO (3.41 m^2^ g^−1^) and tourmaline (12.78 m^2^ g^−1^). Meanwhile, the total pore volume and pore size of the CuO/tourmaline composite (13.744 nm, 0.081 cm^3^ g^−1^), CuO (21.960 nm, 0.019 cm^3^ g^−1^), and tourmaline (15.219 nm, 0.049 cm^3^ g^−1^) were analyzed according to the Barrett–Joyner–Halenda (BJH) method (Figure 4b). The larger pore volume and BET specific surface area confirmed that the CuO/tourmaline composite with 0D/2D CuO structure possessed an increased number of active sites.

**Table 1:** BET specific surface area, total pore volume, average pore size, and average fluorescence lifetime of the samples.

Property	CuO	Tourmaline	CuO/tourmaline

BET specific surface area (m^2^ g^−1^)	3.41	12.78	23.60
total pore volume (cm^3^ g^−1^)	0.019	0.049	0.081
average pore size (nm)	21.960	15.219	13.744
average fluorescence lifetime (ns)	3.24	–	2.94

**Figure 5:**
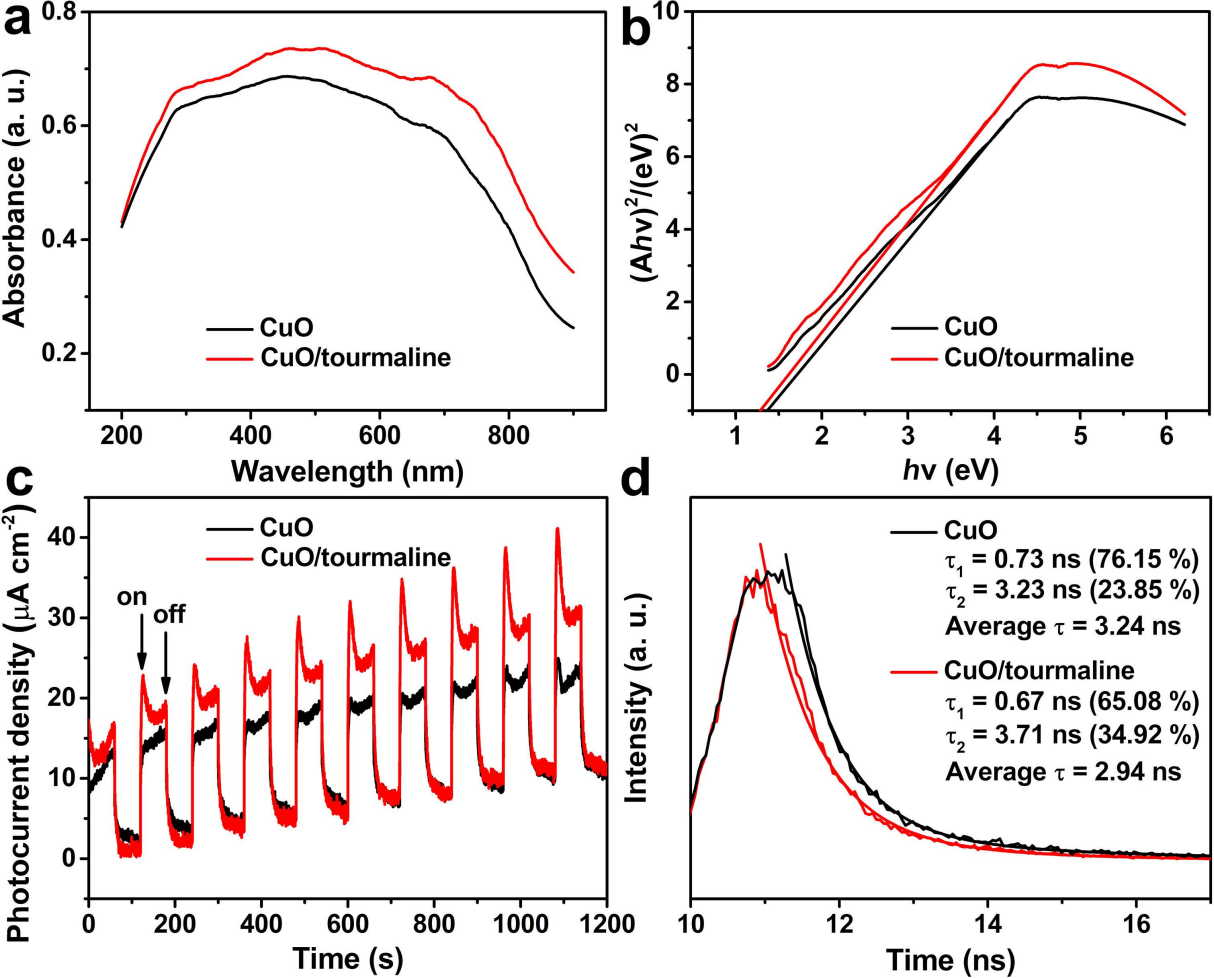
(a) UV–vis diffuse reflectance spectra, (b) plots of (A*h*ν)^2^ vs *h*ν, (c) transient photocurrent, and (d) time-resolved PL spectra of the CuO and CuO/tourmaline composite.

UV–vis diffuse reflectance spectra were collected to study the optical properties of the samples. As shown in Figure 5a, the CuO/tourmaline composite exhibited higher optical adsorption than that of pure CuO, due to the plasmon resonance of the tourmaline units [24]. The band gap of CuO and CuO/tourmaline composite was calculated to be *E*_g_ = 1.38 eV and *E*_g_ = 1.31 eV, respectively (Figure 5b). The lower band gap of the CuO/tourmaline composite suggested a higher utilization efficiency of light [32].

Various measurements were employed to investigate the effect of tourmaline on the separation of photoinduced charge carriers. The transient photocurrent density of the CuO/tourmaline composite was higher than that of pristine CuO during the repeated switching on and off of light irradiation (Figure 5c), implying that tourmaline could facilitate the transfer of photoinduced e^−^, which was also confirmed by the PL spectra (Supporting Information File 1, Figure S1). Generally speaking, the higher the transient photocurrent density, the smaller the electrochemical impedance spectra (EIS) [33–34]. According to the time-resolved PL spectra in Figure 5d, the average fluorescence lifetime of the CuO/tourmaline composite (2.94 ns) was shortened with reference to pristine CuO (3.24 ns) (Table 1). This result indicated that an additional nonradiative decay pathway may be opened through the electron transfer from CuO to tourmaline [35]. The above analyses confirmed the role of tourmaline in suppressing the recombination of photoinduced charge carriers.

**Figure 6:**
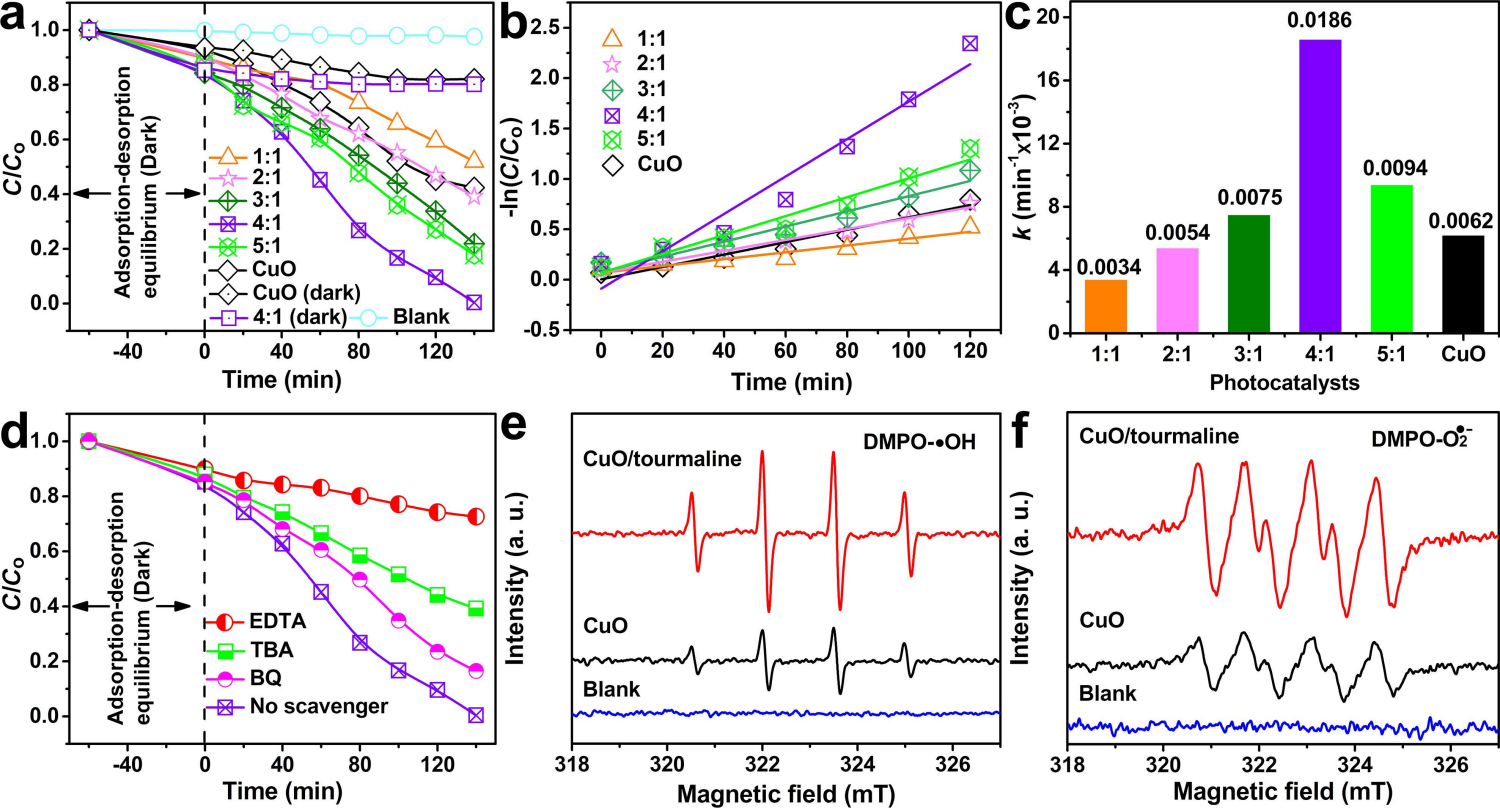
(a) MB degradation, (b) the apparent pseudo-first-order kinetic plots, and (c) rate constant *k* of the CuO/tourmaline composite in different mass ratios of the CuO/tourmaline. (d) MB degradation over the CuO/tourmaline-4:1 composite with different reactive species scavengers. Experimental conditions: [MB]_0_ = 0.01 g L^−1^, [photocatalyst]_0_ = 0.5 g L^−1^, volume = 100 mL, temperature = 25 °C. EPR signals of (e) DMPO-•OH and (f) DMPO-O_2_^•−^ adducts of the CuO and CuO/tourmaline composite under visible-light irradiation.

The photocatalytic degradation of MB was conducted to evaluate the properties of the samples. To assess any real visible-light photocatalytic activity, the adsorption should be excluded [36–39]. Judging from Figure 6a, CuO and CuO/tourmaline-4:1 composite contributed to MB adsorption of 17.9% and 19.8%, respectively. The CuO/tourmaline-4:1 composite exhibited the optimum activity with MB degradation (%) of 99.6% within 140 min. The apparent pseudo-first-order equation was adopted to interpret the photocatalytic degradation kinetics. The corresponding fitted lines of −ln(*C*/*C*_0_) vs time are exhibited in Figure 6b. The *k* value of the CuO/tourmaline-4:1 composite was larger than that of the other composites and was three times higher than that of CuO (Figure 6c), which proved that the CuO/tourmaline composite was successfully prepared and showed higher photocatalytic organic oxidation ability.

Trapping experiments were carried out to verify the predominant reactive species that participated in the photocatalytic organic oxidation reactions. •OH, O_2_^•−^, and h^+^ were trapped by TBA, BQ, and EDTA, respectively [40–41]. Judging from Figure 6d, the addition of TBA, BQ, and EDTA resulted in a decrease in MB degradation from 99.6% to 60.8%, 83.5%, and 27.4%, respectively. The results revealed that h^+^, •OH, and O_2_^•−^ were involved in the photocatalytic reactions, and h^+^ contributed to the higher organic oxidation than •OH and O_2_^•−^. An EPR measurement was used to directly probe the generation of reactive species in the CuO/tourmaline composite aqueous suspensions by using DMPO as a trapping agent. As shown in Figure 6e and 6f, no EPR signals were observed in the absence of light irradiation. When exposed to light irradiation, the intensity of both DMPO-O_2_^•−^ and DMPO-•OH adducts for the CuO/tourmaline composite were stronger than those of pure CuO. The results clearly verified that the CuO/tourmaline composite could produce a higher level of reactive species than pure CuO. The more reactive species the composite produced, the higher photocatalytic activity the composite exhibited.

**Figure 7:**
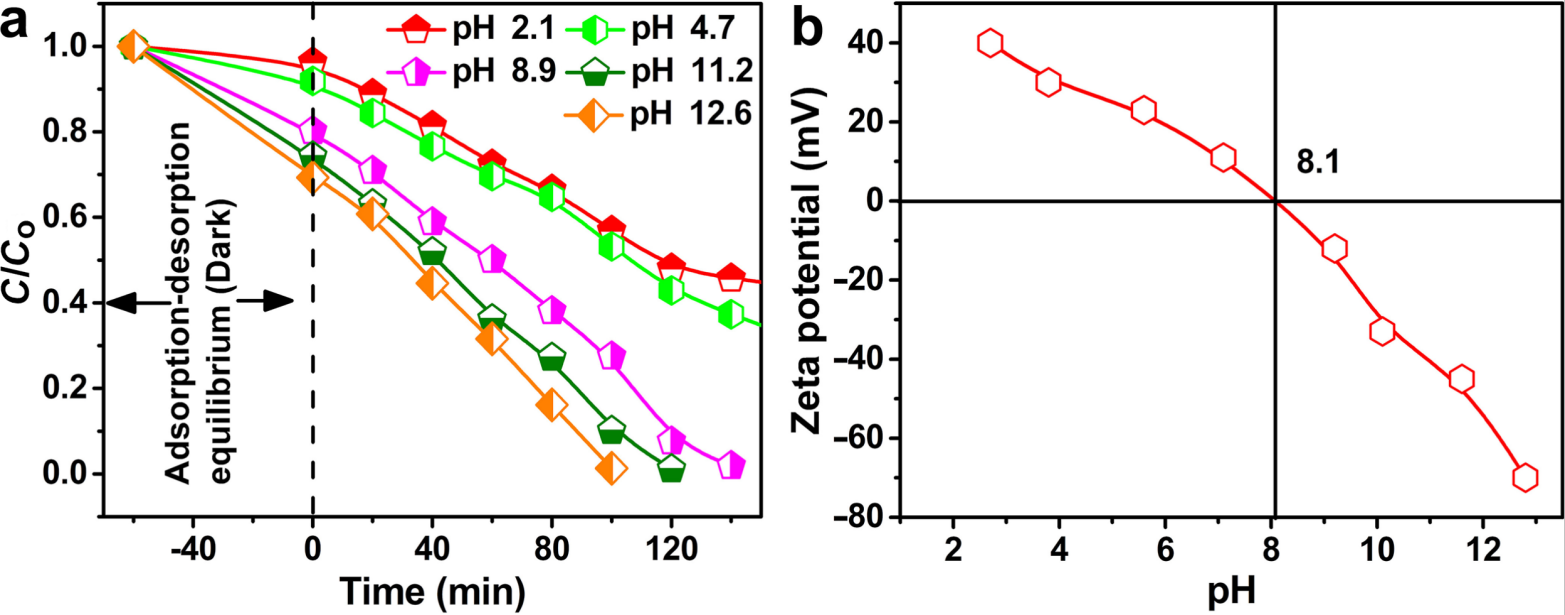
(a) MB degradation over the CuO/tourmaline-4:1 composite with different initial solution pH. Experimental conditions: [MB]_0_ = 0.01 g L^−1^, [photocatalyst]_0_ = 0.5 g L^−1^, volume = 100 mL, temperature = 25 °C. (b) Zeta potential of the CuO/tourmaline composite under various pH values.

The photocatalytic performance of the CuO/tourmaline composite was also tested under different conditions. The composite with the synthesis temperature of 180 °C exhibited optimum photocatalytic activity (Supporting Information File 1, Figure S2), implying that the synthesis temperature of 180 °C could optimize the 0D/2D CuO structure and interfacial interaction between CuO and tourmaline. The initial solution pH affected the photocatalytic performance mainly via changing the adsorption of dye molecules onto photocatalysts (Figure 7a), as adsorption occurred firstly during the photodegradation reactions [42].

The isoelectric point of the CuO/tourmaline composite was determined to be 8.1 (Figure 7b). When the pH value of the solution was less than 8.1, the adsorption between the positively charged composite and the positively charged MB was weakened by electrostatic repulsion. When the pH value of the solution was larger than 8.1, the adsorption between the negatively charged composite and the positively charged MB was strengthened by electrostatic attraction.

**Figure 8:**
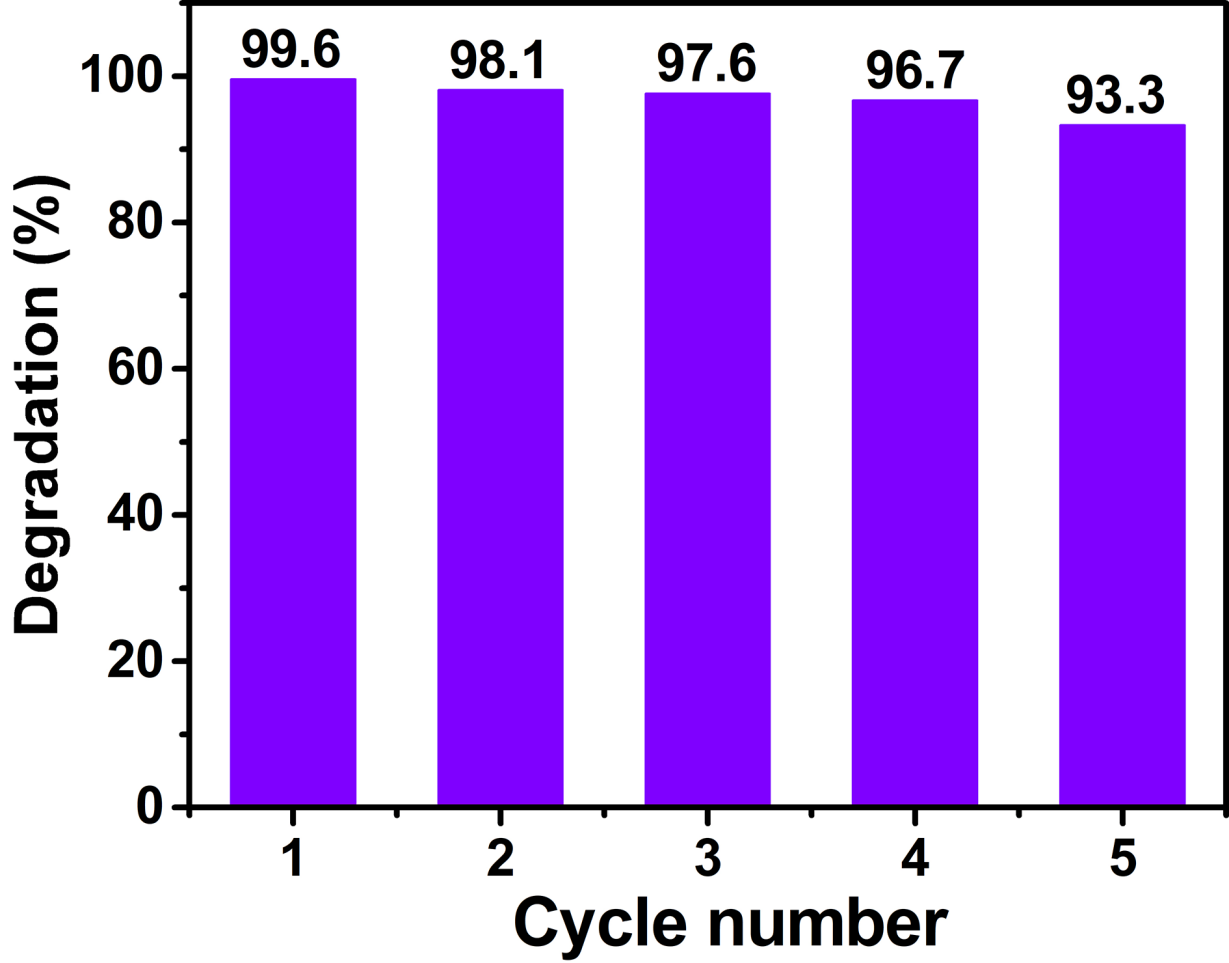
Five successive photocatalytic MB degradation (%) cycles over the CuO/tourmaline-4:1 composite. Experimental conditions: [MB]_0_ = 0.01 g L^−1^, [photocatalyst]_0_ = 0.5 g L^−1^, volume = 100 mL, temperature = 25 °C.

The durability and stability of the CuO/tourmaline composite were evaluated by five successive photocatalytic degradation tests. As shown in Figure 8, MB degradation over the CuO/tourmaline composite remained at 93.3% after being used for five times. There was no obvious distinction between the XRD patterns and FTIR spectra of the fresh and reused CuO/tourmaline composite, except for a slight decrease in the intensity (Supporting Information File 1, Figure S3). The results implied that the as-synthesized CuO/tourmaline composite was durable and stable toward the photocatalytic decomposition of organic pollutants, which might be because tourmaline protected CuO from photocorrosion by facilitating the fast transfer of the photogenerated e^−^.

**Figure 9:**
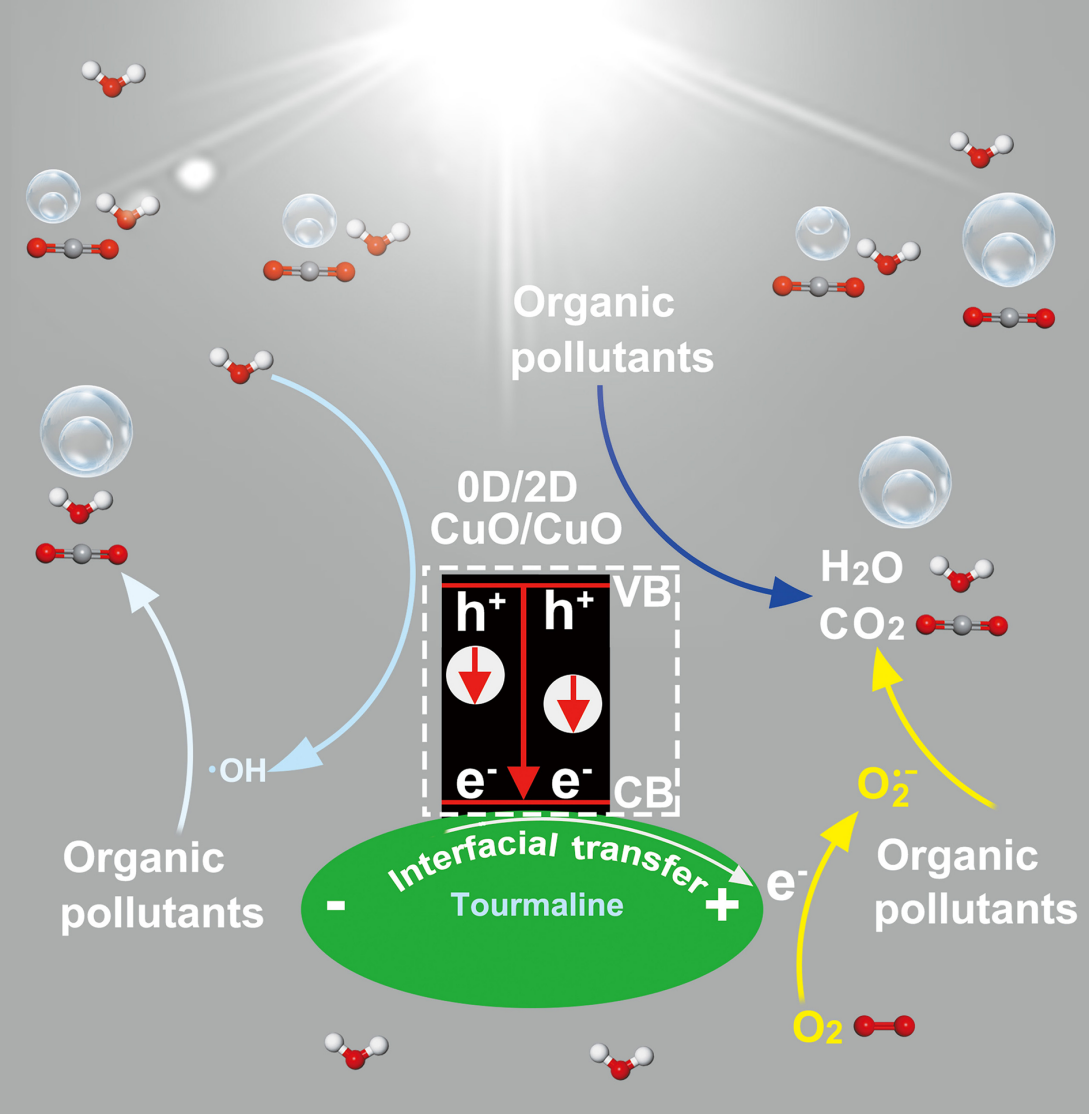
Schematic illustration of the role of tourmaline in enhancing the photocatalytic activity of CuO.

With a combination of the analyses carried out above, a plausible mechanism for strengthening the photocatalytic degradation activity of CuO with tourmaline was proposed and shown in Figure 9. The e^−^ in the valence band (VB) of CuO was excited with the generation of e^−^/h^+^ pairs under light irradiation (Equation 1). Tourmaline facilitate the transfer of photoinduced e^−^ via its strong electric ﬁeld (Equation 2). The separated e^−^ reacted with the oxygen dissolved into water with the formation of O_2_^•−^ (Equation 3). Meanwhile, a portion of h^+^ reacted with water to generate •OH (Equation 4). Reactive species of h^+^, •OH, and O_2_^•−^ synergistically participated in the organic oxidation reactions (Equation 5). The exposure of more adsorption-photoreactive sites and the efficient utilization of photoinduced e^−^/h^+^ pairs enabled by tourmaline contributed to the enhanced photocatalytic activity.

[1] $$\text{CuO}+h\nu \to \text{CuO }({\text{e}}^{-}{}_{\text{CB}})+\text{CuO }({\text{h}}^{+}{}_{\text{VB}})$$

[2] $$\text{CuO }({\text{e}}^{-}{}_{\text{CB}})+\text{tourmaline}\to \text{CuO}+\text{tourmaline }({\text{e}}^{-})$$

[3] $$\text{tourmaline }({\text{e}}^{-})+{\text{O}}_{2}\to {\text{O}}_{2}{}^{\cdot -}+\text{tourmaline}$$

[4] $$\text{CuO }({\text{h}}^{+}{}_{\text{VB}})+{\text{H}}_{2}\text{O}\to \;\cdot \text{OH}+{\text{H}}^{+}+\text{CuO}$$

[5] $$\begin{array}{l} {\text{h}}^{+},\;\cdot \text{OH},\;{\text{O}}_{2}{}^{\cdot -}+\text{absorbed}\;\text{organic}\;\text{pollutants}\\ \to \text{intermediates}\to {\text{CO}}_{2},\;{\text{H}}_{2}\text{O} \end{array}$$

## Conclusion

A CuO/tourmaline composite with 0D/2D CuO architecture was successfully obtained by using a facile hydrothermal process. The CuO/tourmaline composite exhibited superior photocatalytic activity toward organic oxidation, which could be ascribed to the fact that tourmaline can simultaneously enable the formation of 0D/2D CuO to increase the quantity of adsorption-photoreactive sites and promote the effective utilization of photogenerated e^−^/h^+^ pairs. The present study offered a facile strategy for the construction of a 0D/2D CuO structure and the design of tourmaline-based functional composite photocatalysts for the oxidation of organic pollutants.

## Supporting Information

File 1 Additional experimental data.

## References

[1] Ao M, Liu K, Tang X, Li Z, Peng Q, Huang J (2019). Beilstein J Nanotechnol.

[2] Yu C, Tong Z, Li S, Yin Y (2019). Mater Lett.

[3] Gołąbiewska A, Paszkiewicz-Gawron M, Sadzińska A, Lisowski W, Grabowska E, Zaleska-Medynska A, Łuczak J (2018). Beilstein J Nanotechnol.

[4] Helal A, Harraz F A, Ismail A A, Sami T M, Ibrahim I A (2017). Appl Catal, B.

[5] Li W, He S-a, Su Z-y, Xu W, Wang X-c (2019). Appl Surf Sci.

[6] Alp E, Eşgin H, Kazmanlı M K, Genç A (2019). Ceram Int.

[7] Arshad A, Iqbal J, Siddiq M, Ali M U, Ali A, Shabbir H, Bin Nazeer U, Saleem M S (2017). Ceram Int.

[8] Gusain R, Kumar P, Sharma O P, Jain S L, Khatri O P (2016). Appl Catal, B.

[9] Li J-Y, Yuan L, Li S-H, Tang Z-R, Xu Y-J (2019). J Mater Chem A.

[10] de Brito J F, Tavella F, Genovese C, Ampelli C, Zanoni M V B, Centi G, Perathoner S (2018). Appl Catal, B.

[11] Long M, Zhang Y, Shu Z, Tang A, Ouyang J, Yang H (2017). Chem Commun.

[12] Zhu L, Li H, Liu Z, Xia P, Xie Y, Xiong D (2018). J Phys Chem C.

[13] Malwal D, Gopinath P (2016). Catal Sci Technol.

[14] Bharathi P, Harish S, Archana J, Navaneethan M, Ponnusamy S, Muthamizhchelvan C, Shimomura M, Hayakawa Y (2019). Appl Surf Sci.

[15] Shi Q, Ping G, Wang X, Xu H, Li J, Cui J, Abroshan H, Ding H, Li G (2019). J Mater Chem A.

[16] Duan Y (2018). Mater Res Bull.

[17] Chen J, Liu X-P, Yang X-D, Jiang L-L, Mao C-J, Niu H, Jin B-K, Zhang S (2017). Mater Lett.

[18] Khusnun N F, Jalil A A, Triwahyono S, Hitam C N C, Hassan N S, Jamian F, Nabgan W, Abdullah T A T, Kamaruddin M J, Hartanto D (2018). Powder Technol.

[19] Luo X, Li R, Homewood K P, Chen X, Gao Y (2020). Appl Surf Sci.

[20] Wang Q, Chen C, Zhu S, Ni X, Li Z (2019). Res Chem Intermed.

[21] Song Y, Zhu D, Liang J, Zhang X (2018). Ceram Int.

[22] Fu L, Guo Y, Pan S, Huang J, Wang L (2019). Surf Coat Technol.

[23] Li N, Zhang J, Wang C, Sun H (2017). J Mater Sci.

[24] Li K, Chen T, Yan L, Dai Y, Huang Z, Guo H, Jiang L, Gao X, Xiong J, Song D (2012). Catal Commun.

[25] Xue G, Han C, Liang J, Wang S, Zhao C (2014). J Nanosci Nanotechnol.

[26] Ma P, Yu Y, Xie J, Fu Z (2017). Adv Powder Technol.

[27] Makreski P, Jovanovski G (2009). Spectrochim Acta, Part A.

[28] Zhao C, Liao L, Xia Z, Sun X (2012). Vib Spectrosc.

[29] Liu X, Yu C, Yang W, Xie L, Liang S (2019). Eur J Mineral.

[30] Saravanan R, Karthikeyan S, Gupta V K, Sekaran G, Narayanan V, Stephen A (2013). Mater Sci Eng, C.

[31] Umar A, Chauhan M S, Chauhan S, Kumar R, Kumar G, Al-Sayari S A, Hwang S W, Al-Hajry A (2011). J Colloid Interface Sci.

[32] Shu Z, Zhang Y, Ouyang J, Yang H (2017). Appl Surf Sci.

[33] Dong H, Xiao M, Yu S, Wu H, Wang Y, Sun J, Chen G, Li C (2020). ACS Catal.

[34] Li C, Du Y, Wang D, Yin S, Tu W, Chen Z, Kraft M, Chen G, Xu R (2017). Adv Funct Mater.

[35] Cao S, Shen B, Tong T, Fu J, Yu J (2018). Adv Funct Mater.

[36] Li C, Yu S, Dong H, Liu C, Wu H, Che H, Chen G (2018). Appl Catal, B.

[37] Dong H, Zhang X, Li J, Zhou P, Yu S, Song N, Liu C, Che G, Li C (2020). Appl Catal, B.

[38] Li C, Yu S, Che H, Zhang X, Han J, Mao Y, Wang Y, Liu C, Dong H (2018). ACS Sustainable Chem Eng.

[39] Li C, Yu S, Dong H, Wang Y, Wu H, Zhang X, Chen G, Liu C (2018). J Colloid Interface Sci.

[40] Tian N, Huang H, He Y, Guo Y, Zhang T, Zhang Y (2015). Dalton Trans.

[41] Shi L, He Z, Liu S (2018). Appl Surf Sci.

[42] Tan Y, Yu K, Yang T, Zhang Q, Cong W, Yin H, Zhang Z, Chen Y, Zhu Z (2014). J Mater Chem C.

