# Supplementary material for: Synthesis and enhanced photocatalytic performance of 0D/2D CuO/tourmaline composite photocatalysts
Source: Beilstein J Nanotechnol. 2020 Mar 2;11:407–16. doi: 10.3762/bjnano.11.31 (PMC7082706; doi:10.3762/bjnano.11.31)
Supplement: File 1 — Additional experimental data. [file Beilstein_J_Nanotechnol-11-407-s001.pdf]

BEILSTEIN JOURNAL OF NANOTECHNOLOGY

Supporting Information

for

Synthesis and enhanced photocatalytic performance of 0D/2D CuO/tourmaline composite photocatalysts

Changqiang Yu, Min Wen, Zhen Tong, Shuhua Li, Yanhong Yin, Xianbin Liu, Yesheng Li, Tongxiang Liang, Ziping Wu and Dionysios D. Dionysiou

Beilstein J. Nanotechnol. **2020,** *11,* 407–416. doi:10.3762/bjnano.11.31

Additional experimental data

License and Terms: This is a supporting information file under the terms of the Creative Commons Attribution License (https://creativecommons.org/licenses/by/4.0). Please note that the reuse, redistribution and reproduction in particular requires that the authors and source are credited.
The license is subject to the *Beilstein Journal of Nanotechnology* terms and conditions: (https://www.beilstein-journals.org/bjnano)

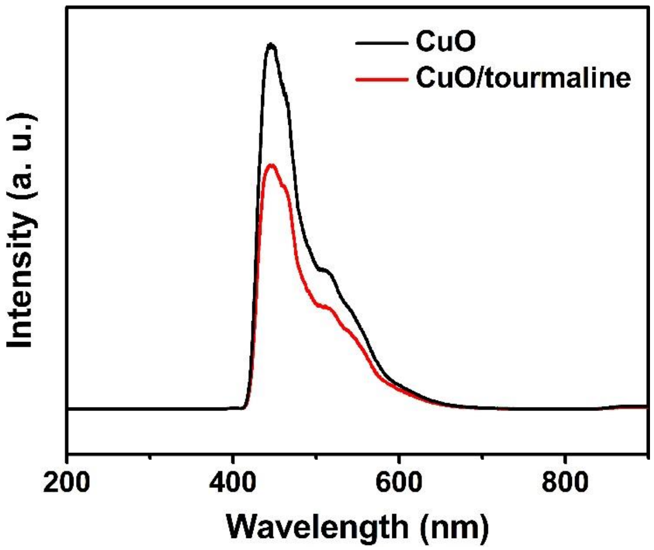

Figure S1: PL spectra of the CuO and CuO/tourmaline composite.

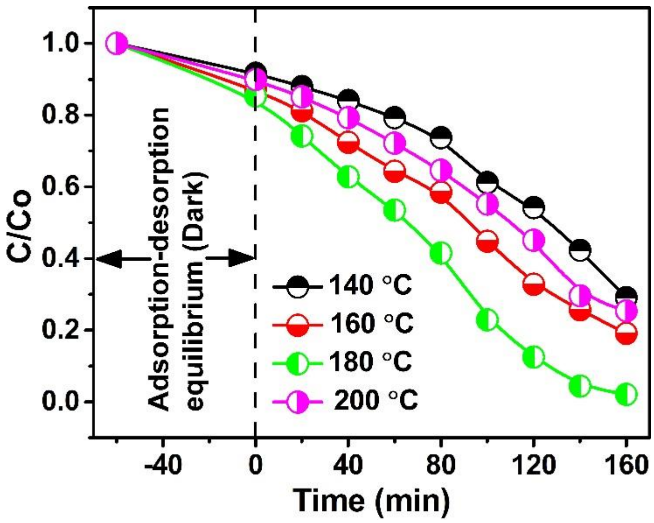

Figure S2: Photocatalytic degradation curves of MB by the CuO/tourmaline composite with different synthesis temperatures.

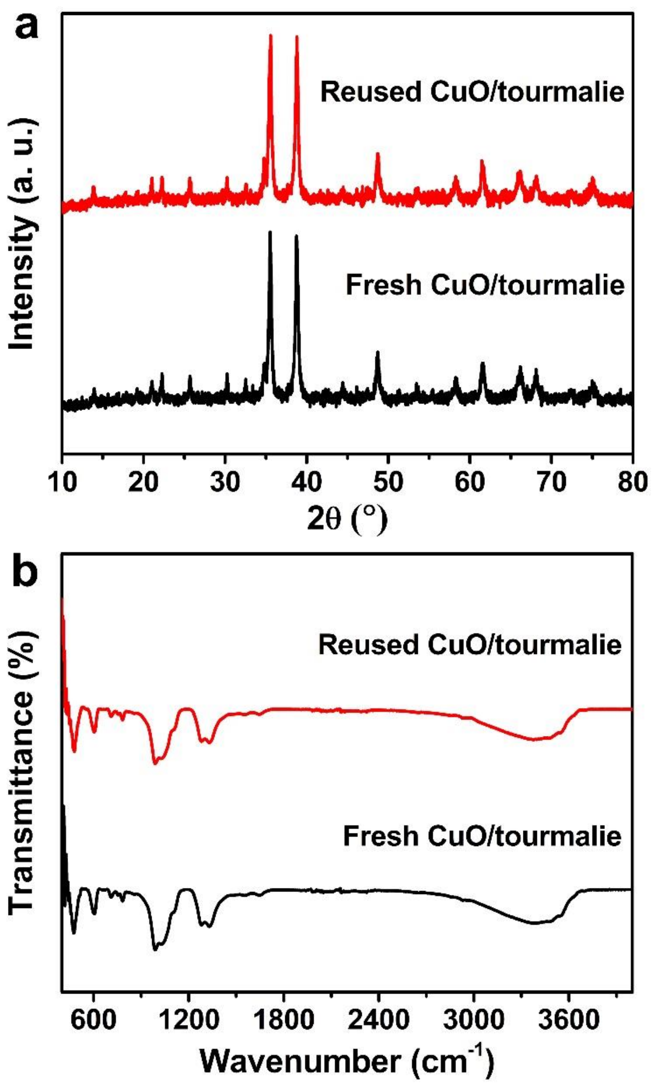

Figure S3: (a) XRD patterns and (b) FTIR spectra of the fresh and reused CuO/tourmaline composite.
